# On the relationships between auditory and visual factors in a residential environment context: A SEM approach

**DOI:** 10.3389/fpsyg.2023.1080149

**Published:** 2023-03-01

**Authors:** Luis Garzón, Luis Bravo-Moncayo, Julián Arellana, Juan de Dios Ortúzar

**Affiliations:** ^1^Facultad de Ingeniería y Ciencias Aplicadas, Universidad Técnica del Norte, Ibarra, Ecuador; ^2^Departamento de Ingeniería Civil y Ambiental, Universidad del Norte, Barranquilla, Colombia; ^3^Grupo de Investigación de Entornos Acústicos, Universidad de las Américas, Quito, Ecuador; ^4^Departamento de Ingeniería de Transporte y Logística, Instituto Sistemas Complejos de Ingeniería (ISCI), BRT+ Centre of Excellence, Pontificia Universidad Católica de Chile, Santiago, Chile

**Keywords:** noise-sensitivity, pleasantness, liveability, SEM, residential environment, immersive environments

## Abstract

To understand the effects of specific elements that may enhance or detract residents’ well-being, it is important to explore the relationships between auditory and visual factors, based on people’s sensory experiences. Although residential environments provide natural experimental conditions to observe these relationships, the complexity of measuring sensory perceptions and their subsequent interpretation constitutes a challenge. This study aims to identify the influence of socio-demographics and residential location characteristics on three latent variables: noise-*Sensitivity*, sound-*Pleasantness*, and visual-*Liveability* in a Latin American city. The methodology is replicable and relies on a digital survey that displays environments in 360-format video and uses sound immersion techniques; it was applied to a sample of household heads in Quito, Ecuador. Based on an efficient experimental design, we selected different residential environments according to acoustic-visual attributes and the proximity to residential, commercial, and recreational land uses. Structural Equation Models (SEM) were estimated using mediating variables. Our results reveal the influence of noise-*Sensitivity* on sound-*Pleasantness* and, indirectly, on visual-*Liveability*. Further analysis shows that the impact of sound and visual perception changes with different socio-demographics and residential location characteristics.

## 1. Introduction

Sounds and visual elements are interdependent in urban environments. In particular, individual perceptions about them tend to vary with socio-demographics and housing location characteristics concerning the neighbourhood and urban services (Sun et al., 2018a). However, most applied research on this subject has gravitated towards the idea of controlling noise annoyance by reducing sound pressure levels to enhance well-being (EEA, 2020). Beyond the noise abatement approach, it has been highlighted that audio-visual elements may be subjectively measured to identify improvements in urban settings (Hasegawa and Lau, 2022). This requires the collection of individual perceptions on site, but implementing a methodology to control the stimuli, and collect information from multiple locations at a time, represents an experimental challenge (Tarlao et al., 2021). Moreover, integrating complex path models for exploring the influence of objective attributes amongst subjective factors also requires sophisticated analytical modelling tools (Lionello et al., 2020).

Regarding the study of sounds in the urban environment, the soundscape series of ISO (2014) provides a conceptual framework for acoustic and sound-related research. A growing number of auditory factors are revealed in the literature (i.e., noise annoyance, pleasantness and eventfulness) and used to evaluate the conditions of the acoustic environment (Aletta et al., 2016). Traditionally, experiments designed to analyse residential selection have aimed to discover the influences between noise-sensitivity and noise-annoyance, using socio-demographic characteristics as mediation factors (Miedema and Vos, 2003; Van Kamp et al., 2004; Nijland et al., 2007). However, there has been recent interest in the analysis of auditory factors (e.g., pleasantness and eventfulness) and person-related attributes (noise annoyance) in a residential context, as well as using mediating pathways concerning socio-demographic characteristics (Tarlao et al., 2021).

The evidence reviewed about the perception of visual factors studies have demonstrated its association with numerous concepts such as liveability, satisfaction, and happiness (Ahmed and El-Halafawy, 2019). These concepts play a crucial role when studying the enhancement of the residential environment (Kovacs-Györi et al., 2019). Mouratidis and Yiannakou (2022) recently asked what makes cities liveable and explored how objective and subjective measures may be used to assess the liveability conditions of a place. These measures tend to differ according to the world’s regions because citizens and societies have different wealth and degrees of accessibility to public services (UN, 2022). Thus, urban liveability conditions have become an important topic to be subjectively investigated as a qualifying factor, as well as to be objectively measured. However, in the Latin American region, only a few studies have attempted to capture measures of subjective indicators such as liveability (i.e., by responding to survey questions where audio-visual stimuli are reproduced on the senses of participants), for subsequent interpretation using other perceptual factors (Kogan et al., 2017; Rey-Gozalo et al., 2018).

A systematic process to interpret the relationships amongst the perceptual factors and their corresponding indicators has required the application of sophisticated analytical tools, including advanced machine learning techniques, but the focus has been on the use of multivariate techniques such as the Structural Equation Models (SEM) approach. Applied complex models using SEM have involved perceptual factors of interest (i.e., auditory and visual factors), and include mediating effects through the specification of quantitative attributes (Hong and Jeon, 2015; Liu et al., 2019; Zhao et al., 2021). However, we are not aware of prior statistical estimations using a combined specification between *Pleasantness* and *Liveability* in a residential environment, when exploring direct and indirect effects amongst objective attributes.

This study explores the relationships between three latent variables (i.e., noise-*Sensitivity*, sound-*Pleasantness* and visual-*Liveability*) based on experimental conditions in a selection of residential environments. For this, audio-visual stimuli were recorded in widely dispersed residential locations selected to match different configurations of attributes defined under an efficient experimental design. Audio-visual stimuli were reproduced using immersive devices (360°-format video and immersive sound) and applied to a sample of household heads, collecting perceptual indicators about their residential locations using a digitally assisted survey format. Finally, SEM was estimated to understand the behaviour of the latent variables when modifying quantitative measures of visual and acoustic attributes, socio-demographics and housing location characteristics.

The rest of the paper is organised as follows. The second section briefly reviews our theoretical auditory and visual factor evaluation framework. The third section describes the hypothesis statements and the survey design process methodology. The fourth section contains modelling results for an Exploratory Factor Analysis (EFA), Confirmatory Factor Analysis (CFA), SEM structure and subsequent mediation analysis. The fifth section presents a discussion, and the sixth summarises our main conclusions.

## 2. Theoretical framework

In terms of evaluating the acoustic environment, urban studies have traditionally been concerned with assessing perceptions such as noise-annoyance (Tillema et al., 2012). However, several auditory indicators have emerged as a part of the soundscape concept, providing a broader understanding of improvements in acoustic environments in residential settings (Aletta et al., 2016). There is a growing body of literature using this framework to investigate how contextual indicators (person-related characteristics and non-auditory contextual indicators) influence both auditory indicators (i.e., perceived sources and sound indicators) and soundscape factors (Hong and Jeon, 2015; Liu et al., 2019; Zhao et al., 2021; Hasegawa and Lau, 2022). The notion of auditory factors, especially pleasantness, has “proved particularly relevant to assess the quality of the sound environment” (Aumond et al., 2017, pp. 431). The literature highlights the study of pleasantness based on the selection of a number of auditory indices defined in the Circumplex Model of soundscape perception (Axelsson et al., 2010). The model uses four bipolar factors that can be synthesised by specifying two latent constructs, *Pleasantness* and *Eventfulness* (two main orthogonal factors). Aletta et al. (2016) suggest that the former is the main factor analysed in soundscape studies, and several indicators focused attention on its identification, such as *agitating-calm*, *interesting*, *pleasant–unpleasant*, *appropriateness*, *harmonious* and *comfortable–uncomfortable*.

In addition, a growing literature has looked at liveability (Campbell et al., 1976; Van Kamp et al., 2003), although we are not aware of previous modelling approaches selecting subjective indices to analyse it. In European cities, liveability has been associated with indices measuring the quality of life (Marans, 2012; Okulicz-Kozaryn and Valente, 2019). However, Mouratidis and Yiannakou (2022) argue that “liveability can be assessed in a subjective way,” if the term is understood and measured as visual perception (Veenhoven, 2000; Rossetti et al., 2019; Türkoğlu et al., 2019; Ramírez et al., 2021). On the other hand, Ahmed and El-Halafawy (2019) suggested that the perception of visual factors may be associated with concepts such as liveability, satisfaction and well-being. So, these concepts may play a crucial role when studying enhancements to the residential environment (Kovacs-Györi et al., 2019).

Several approaches have used SEM to discover relationships between pleasantness and eventfulness but also specify visual constructs such as satisfaction and visual quality (Mouratidis, 2020; Mouratidis and Yiannakou, 2022). However, to the best of our knowledge, none has examined and quantified the relationship between *Pleasantness* and *Liveability*, and only a theoretical framework has been developed (Aulia, 2016; Amir et al., 2021). This lack of knowledge is even more notorious in the Latin American context, as only a few studies have attempted to conceptualise multidimensional sensory indicators when exploring residential environments (Kogan et al., 2017, 2018; Rey-Gozalo and Barrigón-Morillas, 2017; Rey-Gozalo et al., 2018).

From a methodological perspective, experiments conducted to evaluate the conditions of the urban environment highlight the importance of determining the roles of audio-visual attributes and their effects on individual perceptions (Hong et al., 2020; Yong et al., 2021). The variation of sound and visual–spatial metrics stimulates different feelings in people, and their relationship may enhance well-being in residential environments (Hasegawa and Lau, 2022). However, it is complex to recreate different urban configurations to evaluate audio-visual conditions in an experimental setting with appropriate trade-off levels (Ortega et al., 2020; Ramírez et al., 2021). Recently, access to new technological tools has facilitated the recording and reproduction of visual features (i.e., people and vehicles, green spaces and the built environment) in terms of dynamics, position in space, dimensions and even colours (Puyana-Romero et al., 2016; Arellana et al., 2020). In particular, the use of immersive environments and digital format questionnaires has allowed a more significant number of scenarios to be assessed, allowing for a larger number of data points to be collected simultaneously. However, beyond the overall flexibility provided by these technological tools for survey implementation, some other issues need to be addressed, such as the reproduction length of the experiment, when audio-visual stimuli are projected on the senses of participants and the way to capture the responses. An inadequate method for experimental reproduction can lead to boredom, fatigue, and a high cognitive load on respondents (Heggie et al., 2019).

## 3. Materials and methods

This section describes the characteristics of the participants in the study, the survey design and the statistical tools used to analyse the data.

### 3.1. Participants

Five hundred and forty-three household heads participated in this study. The participants were selected according to the socio-demographic characteristics of the population by groups of sex and age (trends from the latest 2010 national census). The information was collected from November 2020 to June 2021 in 31 urban districts distributed in three main areas of the Metropolitan Area of Quito (i.e., North, Centre and South). Table 1 summarises the main information collected from participants.

**Table 1:** Socio-demographic information (*N* = 543).

Age	Total (%)	Level of education	Total (%)	Impairment problems	Hearing (%)	Visual (%)
26–35	225 (41%)	Not specified	3 (0.6%)	Eyeglasses	-	195 (36%)
36–45	132 (24%)	Undergraduate	116 (21.4%)	Unknown^*^	11 (2%)	22 (4%)
46–55	108 (20%)	Bachelor	67 (12.3%)	None	532 (98%)	326 (60%)
56–65	61 (12%)	University	276 (50.8%)			
65–70	17 (3%)	Master	59 (10.9%)			

* Unknown refers to participants who stated that they were unaware of any problem impairment.

### 3.2. Factor and attribute selection

Our aim was to measure multisensorial factors regarding different residential environment locations. Specifically, the following factors were assessed: (i) noise-*Sensitivity* described as *η_1_*, (ii) sound-*Pleasantness,* labelled as *η_2_*, and (iii) *Liveability*, listed as *η*_*3*,_ to explore visual characteristics in the surroundings of a residence. The factor selection process and corresponding indicators are described in the following sub-section. The statements associated with each perceptual indicator resulted from an extensive literature review and subsequent pilot surveys applied to evaluate their appropriateness according to the experimental context, as shown in Table 2. To measure the indicators, we used a five-point Likert scale (1 = strongly disagree to 5 = strongly agree). The applied survey also included a questionnaire to collect data on sound-visual measures, socio-demographics and dwelling location characteristics, as these attributes could be correlated with the multisensorial factors.

**Table 2:** Factors, indicators, and variables.

Variable/factor	Question/indicator	Scale
Socio-demographics (household head)		
	Gender	Binary
	Age	Categorical
	Occupation	Categorical
	Health insurance	Categorical
	Level of education	Categorical
	Family income	Categorical
	Health impairment declaration	Categorical
Residential location characteristics		
	District of residence	Categorical
	Dwelling type and type of ownership	Categorical
Noise-Sensitivity $\left(\eta_{1}\right)$		
	I1. I am easily awakened by noise (*easily awakened*)	Five-point Likert
	I2. I find it difficult to relax in a noisy place (*relaxation*)	Five-point Likert
	I3. I complain to people who make intense sounds that keep me from falling asleep (*complain*)	Five-point Likert
	I4. I am sensitive to noise (*sensitivity*)	Five-point Likert
Sound-Pleasantness $\left(\eta_{2}\right)$		
	I5. Sounds in this area are pleasant to hear in the surrounding of a dwelling (*pleasant*)	Five-point Likert
	I6. Sounds in this area transmit calm to the surrounding houses (*calm*)	Five-point Likert
	I7. Sounds of this place transmit harmony to inhabitants (*harmonious*)	Five-point Likert
Visual-Liveability $\left(\eta_{3}\right)$		
	I8. This residential neighbourhood is attractive to live in (*attractive*)	Five-point Likert
	I9. I could live in a dwelling located in the surroundings of this place (*liveable*)	Five-point Likert
	I10. In this place, I feel safe from crime (*safe*)	Five-point Likert

#### 3.2.1. Noise-sensitivity perception

Insights into the effects of noise-sensitivity and noise-annoyance have been traditionally explored in urban environments (Nijland et al., 2007). We extend this line of research by investigating how noise-sensitivity impacts auditory factors, by measuring individual perceptions and how they change according to person-related characteristics (Tarlao et al., 2021). Noise-*Sensitivity* can be measured using different questionnaires, including six items (Kishikawa et al., 2006), 10 items (Zimmer and Ellermeier, 1999) or 21 items (Weinstein, 1978). Following Kishikawa et al. (2006), we selected four items from their six-item questionnaire to measure noise-*Sensitivity* in residential environments.

#### 3.2.2. Sound perception

According to the Circumplex Model of Axelsson et al. (2010), at least two orthogonal descriptors are required to achieve a comprehensive understanding of the acoustic environment. However, we specified sound-*Pleasantness* (*η_2_*) as a single latent variable, representing only a part of the aspects investigated for the soundscape. We attempted to build a model keeping a parsimonious structure by using only one factor. Note that *Pleasantness* in outdoor conditions already explains 50% of the variance of the urban soundscape (Axelsson et al., 2010). Hence, we sought to discover the causal relationships between *Pleasantness*, socio-demographic characteristics, and the residential location of the participants. The selection of the indicators *pleasant* (I5), *calm* (I6), and *harmonious* (I7), was guided by the estimation of the highest factor loading amongst those reported by Tarlao et al. (2021), Liu et al. (2019), and Hong and Jeon (2015). Further analysis showed that these three adjectives were highly correlated with *Pleasantness* and jointly described the positive characteristics of sounds.

#### 3.2.3. Visual perception

Visual*-Liveability* (*η_3_*) was selected as the latent construct to evaluate visual conditions. Several perceptual indicators can measure this factor, such as *aesthetics* (Bonaiuto et al., 1999), *habitable* (Veenhoven 2000), *attractive* (Türkoğlu et al., 2019), and *satisfaction* (Mouratidis and Yiannakou, 2022). In this study, the selected indicators refer to issues previously evaluated in Latin American cities, such as *attractive* (I8), *liveable* (I9), and *safe* (I10). The first two are associated with the positive physical neighbourhoods’ qualitative attributes, which vary according to physical and natural components (Rossetti et al., 2019; Ramírez et al., 2021). *Safe*, on the other hand, can affect people’s overall liveability perception and is closely related to the presence of people and vehicles in the city (Iglesias et al., 2013).

#### 3.2.4. Socio-demographic and residential location characteristics

The socio-demographic information was consistent with the variables included in Ecuador’s population censuses and complemented using household information such as the residential location.

#### 3.2.5. Survey experimental design

Instead of recreating sound-visual stimuli artificially, we recorded several real-life scenarios to represent different levels of sound-visual attributes. Five sound-visual attributes associated with residential environments were selected (see Table 3) following the results of previous studies (Jo and Jeon, 2020; Yong et al., 2021). In particular, the *Sound Pressure Level* (SPL) was used and classified as lower and higher than 70 dbA (Yang and Kang, 2005). The *green space* attribute was measured and classified into lower and higher than 0.25 ha using the imagery available on Google Maps. The variables *pedestrian-flow* and *vehicular-flow* were measured on-site and put into two categories: pedestrian-flow lower and higher than 50 (ped/min/m), and vehicle-flow lower and higher than 18^1^ (veh/min/lane; Transportation Research Board, 2010). Finally, land uses were categorised to discover the influences in the residential location of proximity to residential, commercial, and recreational environments.

**Table 3:** Attributes considered in the experiment.

Attribute	Description	Levels
Equivalent SPL(L_Aeq_)	dBA	[less than 70] Moderate and High [more than 70]
Green space (*GS*)	Hectares	[less than 0.25] Low and High [more than 0.25]
Pedestrian flow (*PF*)	ped/min/m	[less than 50] Low and High [more than 50]
Vehicle flow (*VF*)	veh/min/lane	[less than 18] Low and High [more than 18]
Land uses (*LU*)		Residential, commercial, and recreational

**Figure 1:**
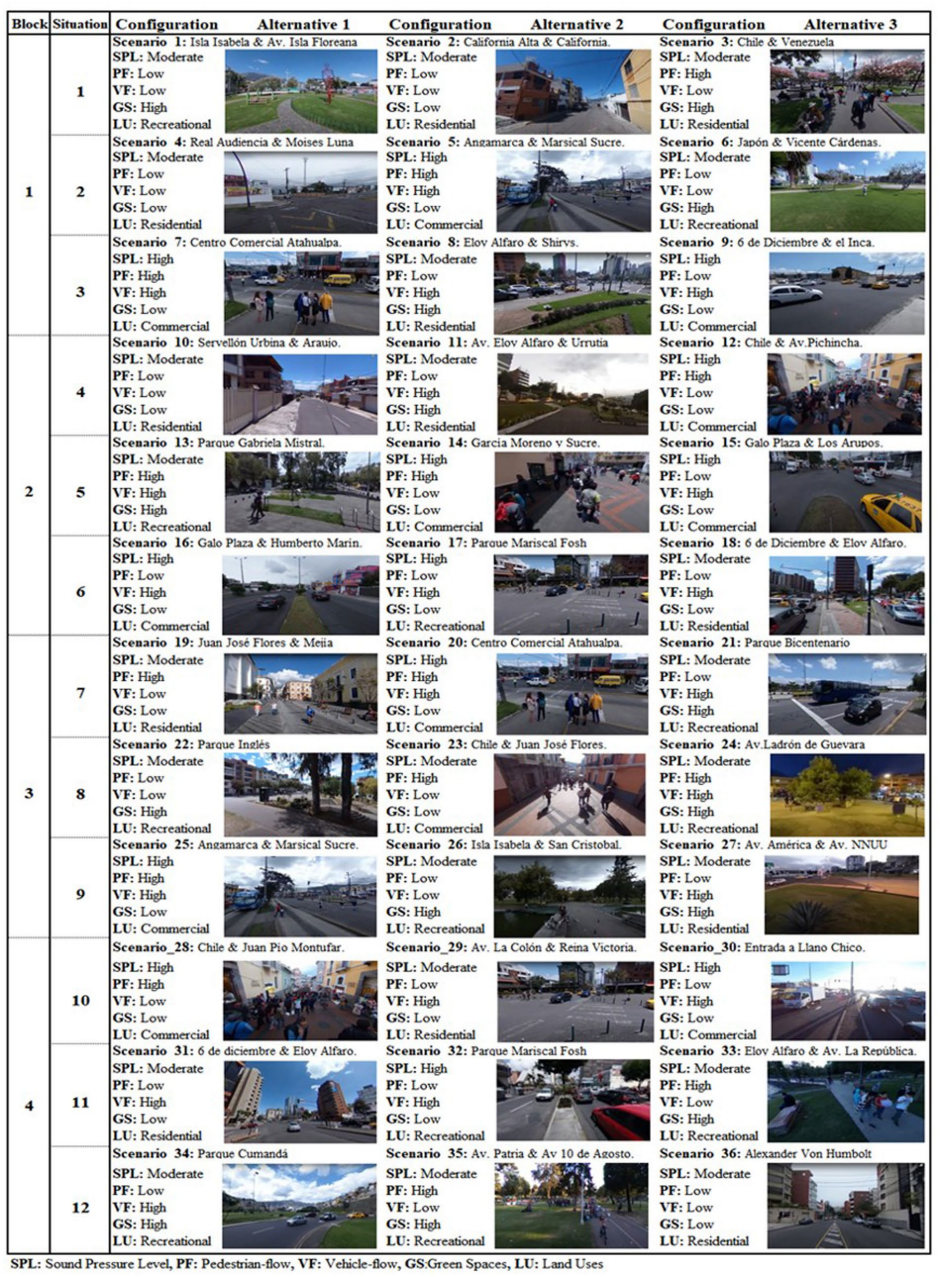
Residential environment scenarios.

Using the above attributes, a D-efficient experimental design (Rose et al., 2008) using the NGENE software allowed us to configure 36 residential scenarios. As shown in Figure 1, the residential surroundings locations were selected according to the experimental design and the noise map of Quito (Bravo-Moncayo et al., 2019), which is categorised according to SPL.

#### 3.2.6. Sound and visual stimuli

After identifying the residential environment of the different Quito districts, we measured and collected audio-visual extracts *in situ*. Then, two research group members observed the 36 recordings separately to ensure consistency and correspondence to the category specified on each attribute, as described in Table 3.

Once the location was identified, 1-min auditory samples were recorded *in situ* using a portable four-channel cardioid Ambisonic 3D TA-1 microphone, which captured the natural directionality of the spatial audio. Afterwards, these stimuli were used in a playback setup. A second microphone was connected simultaneously with a portable sound level metre (NTI-Audio Model-XL2) for acoustic measurement, as described in Table 4. Both microphones were installed at 1.6 m from the ground to approximate the height of a person’s ears when standing.

**Table 4:** Sound-visual value measurements.

Scenario	L_Ceq,60s_ dB	TSLV dB	L_10_ dB	L_90_ dB	L_10−_L_90_ dB	L_Aeq,60s_ dB	Pedestrian ped/min/m	Vehicles veh/min/lane	Green spaces hectares	Link (YouTube) https://youtu.be/
1	67	1.47	52.5	46.7	5.8	50.3	8	7	0.30	cmExi8ApWD4
2	70.6	4.83	60.5	44.1	16.4	58.7	3	5	0.01	Ncuqh7GbVhw
3	69.6	0.99	63.3	58.0	5.3	61.2	>99	1	0.30	_SzT2on7SHY
4	68.6	10.61	63.3	50.5	12.8	58.8	4	9	0.01	0nA9a2We36M
5	84.8	5.56	74.2	66.9	7.3	73.1	53	41	0.06	Vv1ifivpKbY
6	72.2	0.4	60.8	57.8	3.0	59.5	14	4	0.40	L7J6SkIOlhY
7	84.6	16.11	79	65	14.0	74.5	91	42	0.17	eLcCJ1GCN_c
8	76.8	15.96	67.8	54.4	13.4	63.8	9	77	>0.99	uSKB1JT0CKk
9	97.1	3.5	76.2	66.2	10.0	72.7	9	>99	0.02	3zJTAPv_6yM
10	78.1	6.22	67.1	61.7	5.4	64.6	1	1	0.01	Y6Q18O-7zVs
11	83.9	5.57	68.9	60.2	8.7	55.4	1	>99	0.53	4 g-flhXB_S8
12	83	1.48	80.8	73.7	7.1	77.9	>99	3	0.01	Xi6Rf2vCbF0
13	76.5	0.81	70.7	63.5	7.2	68.6	70	58	0.35	K-Ual5evQi0
14	79.2	3.74	76	68.8	7.2	73.6	>99	8	0.01	teTZ6hRLrdc
15	87.7	4.39	81.3	74.9	6.4	78.5	8	>99	0.01	r1UZnNOaq98
16	84.4	8.73	78.9	72.3	6.6	78.9	6	>99	0.20	eTckO6zJHOA
17	86.9	4.79	74.8	65.9	8.9	73.1	8	21	0.01	rrPub_afPhk
18	83.6	10.62	71.7	63.3	8.4	68.4	29	72	0.10	krew6cil3FM
19	72.9	0.31	68.9	64.7	4.2	67.1	52	4	0.01	-Kjyh55qsFA
20	86	4.64	79.3	68.9	10.4	76.5	91	42	0.17	eLcCJ1GCN_c
21	81.5	2.09	67.1	61.5	5.6	64.5	5	18	0.99	HNzlrihiV-o
22	67.1	1.47	57.5	51.2	6.3	54.2	5	1	>0.99	GCSYAeuJXwM
23	79.4	3.77	70	63.4	6.6	67.5	52	7	0.01	Qxx9m0FLFfE
24	76.5	0.81	70.7	63.5	7.2	68.6	51	58	0.40	DjChFYcVet0
25	84.8	5.56	74.2	66.9	7.3	73.1	89	51	0.01	Vv1ifivpKbY
26	65.3	3.36	57	47.1	9.9	53.2	4	6	0.99	MNwvuKQVrSM
27	81.8	1.06	70	66.4	3.6	68.6	17	98	0.39	Raet_pTkY1E
28	83	1.48	80.8	73.7	7.1	77.9	84	11	0.01	Xi6Rf2vCbF0
29	80.4	3.51	69.9	59.3	10.6	66.4	39	63	0.01	llzO18JpsgU
30	88.1	10.57	79.2	69.5	9.7	76.4	7	>99	0.01	6W134ASMQwo
31	83.6	10.62	71.7	63.3	8.4	68.4	29	72	0.10	krew6cil3FM
32	86.9	4.79	74.8	65.9	8.9	73.1	8	21	0.01	rrPub_afPhk
33	67.5	0.18	61.2	58.8	2.4	60.2	38	1	>0.99	-G0RVZKco-I
34	78.8	2.58	70.7	63.5	7.2	68.9	6	79	>0.99	cZ0R4Z2Ja_E
35	70.4	3.18	64.3	54.8	9.5	69.6	76	1	>0.99	KEQ7uNgW3K0
36	68.6	10.61	63.3	50.5	12.8	58.8	1	5	0.01	cnTxJfG1BUQ

The equivalent A-weighted (L_Aeq,60s_) and C-weighted (L_Ceq,60s_) continuous sound pressure levels were measured. Then, its difference was calculated in dB (L_Ceq,60s_-L_Aeq,60s_) to indicate the relative proportion of low-frequency sounds. Acoustic measurements of the SPL of the 10th percentile were also obtained (L_10_), representing the most energetic noise sources exceeding 10% of measurement time. In addition, the Temporal Variance of the Sound Pressure Level (TSLV), indicating the variability of the SPL over time during the measurement, was also estimated. The range L_A10_–L_A90_ was also used as an index of sound environment variability, and it denotes the difference between percentile levels exceeding 10 and 90% of the time.

Complementing the sound recordings, visual stimuli were also obtained using a spherical panoramic camera with 4 k ultra-high definition (RICOH THETA V) mounted at 1.6 m from the ground to capture omnidirectional video at each location. All videos were recorded for 60 s. Each excerpt was processed as a 360-format video in combination with the spatial audio format. This method was described by Hong et al. (2017) and recently applied to recreate artificially audio-visual stimuli conditions (Hong et al., 2020; Yong et al., 2021). However, in this research, all scenarios were recorded to capture the real conditions in different residential surroundings and then reproduced for experimental purposes. All scenarios were captured regarding daytime and avoiding rainy conditions (from 09:00 a.m. to 5:00 p.m.). The immersive audio-visual excerpts were uploaded to the YouTube platform to be displayed on any device that supports 360 format videos; the links are presented in Table 4, together with the values of the acoustic measurements.

### 3.3. Experimental setup and exposure conditions

The survey was issued in a digital format and comprised three sections:

1. Socio-demographic and location information (see Table 2).
2. Perceptual indicator measurements (see Table 2). In this section, the 36 scenarios were grouped into four blocks of nine scenarios. Then, each participant observed the reproduction of nine scenarios according to the experimental design (see Figure 1). Note that data gathered from each participant correspond to the level of agreement with all indicators measuring noise-*Sensitivity*, sound-*Pleasantness*, and visual-*Liveability* for every situation within one block.
3. A stated preference experiment containing three choice situations to assess location preferences. This last section is not used in this paper.

**Figure 2:**
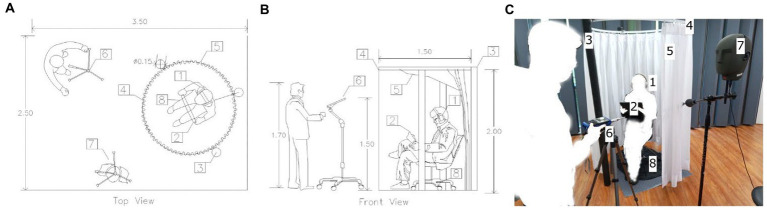
Top view **(A)**, front view **(B)**, and photo **(C)** of the experimental setup **(C)**. Details of equipment and portable structure (1) headphones, (2) tablet, (3) columns, (4) curtain rails, (5) curtain, (6) sonometer, (7) dummy head, and (8) swivel chair.

The experiment was conducted under isolated conditions using a portable round cabin (see Figure 2). Three columns of the cabin were used to support a curtain attached to a rail. This configuration was intended to isolate respondents from external distractions during the experiment administration. Before entering the cabin, each participant was instructed to use a 13-in digital tablet screen and noise-cancelling headphones, as shown in Figure 2. The SPL was also measured as a part of the experiment setup applications. On average, it took between 25 and 30 min to complete each survey, including viewing time.

Our approach offers the advantage of portability for conducting the survey. In addition, its cost is lower than the investment needed to use a laboratory, according to the respondents’ schedules. We had also planned to use a head-mounted virtual reality device (VR-HMD). However, its implementation was complex, and the internet connection required for its operation was not always available on-site. Therefore, a tablet with mobile data and internet support was chosen to administer the survey. The ecological validity of visual devices has been evaluated previously (Sun et al., 2018b; Hong et al., 2020), as well as applying digital surveys formats in different fields of knowledge (Liebe et al., 2015; Arellana et al., 2020; Weber et al., 2022).

### 3.4. Data analysis

First, we performed an exploratory factor analysis (EFA) to extract the main latent constructs from a set of measured indicators without a preconceived structure. Once the structure between indicators and latent variables was identified, a second step based on CFA was applied. The CFA tests the reliability and validity of measurement scales for observed and latent variables. Next, an advanced SEM model was formulated to validate the hypothesis path by estimating the regressor coefficient loadings on the structural model. SEM models were processed and analysed using the Lavaan library (Rosseel, 2012) in R-Studio (CoreTeam, 2018). Finally, SEM allows the use of mediation analysis and then estimates the loadings of the total effects involving both latent and explanatory variables.

## 4. Results

The results are presented in five sections: (1) a descriptive statistical analysis of collected indicators; (2) the EFA to extract principal factors; (3) the CFA to examine the reliability and validity of latent variables; (4) the SEM to describe the relationships amongst the latent constructs; (5) and the mediation analysis to estimate the influence of observable variables on the latent constructs.

### 4.1. Descriptive analysis of indicators

**Figure 3:**
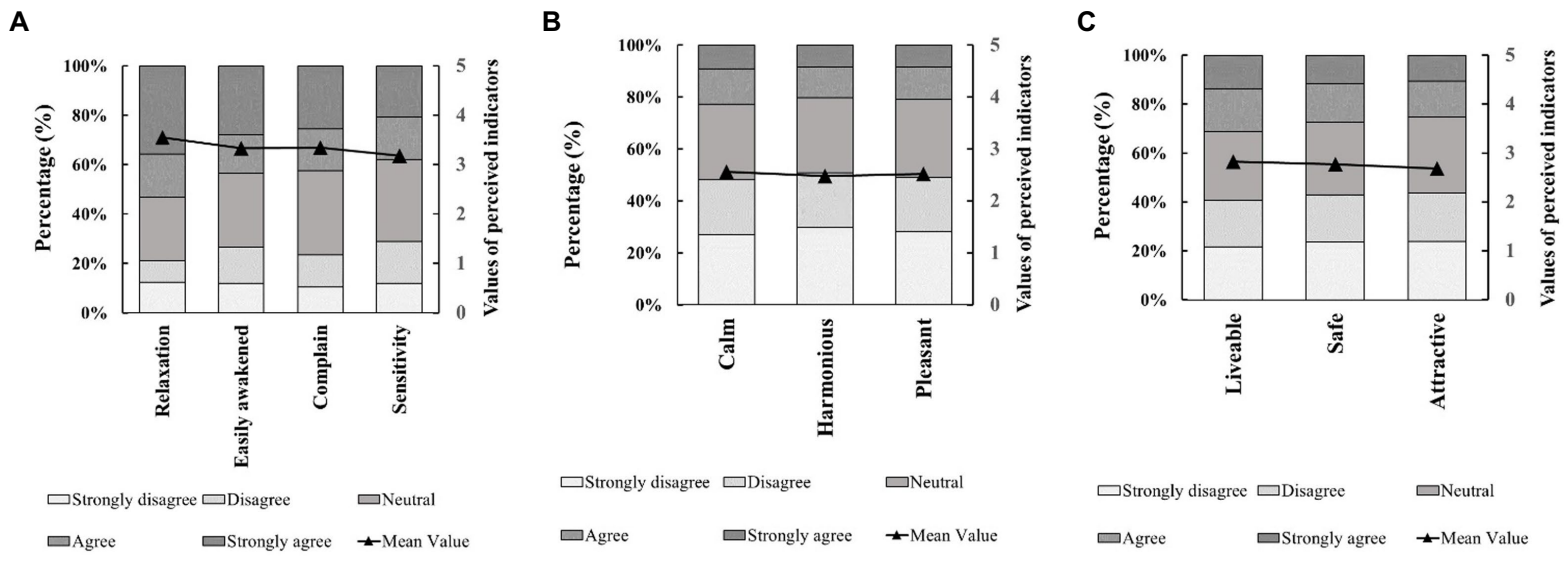
Rates of perceived indicators of noise-sensitivity **(A)**, sound-pleasantness **(B)**, and visual-liveability **(C)**.

Figure 3 summarises the responses to the perceptual indicators described in Table 2. The averages for *relaxation* (I2), *easily awakened* (I1), *complaint* (I3) and *sensitivity* (I4), related to noise-*Sensitivity*, show high perceived values (3.55, 3.33, 3.34 and 3.18, respectively). The mean values for sound-*Pleasantness* indicators, such as *calm* (I6), *harmonious* (I7) and *pleasant* (I5), are relatively neutral (2.57, 2.48 and 2.52, respectively), and the same happens with the visual-*Liveability* indicators, that is, *liveable* (I9), *safe* (I10) and *attractive* (I8), with values of 2.83, 2.77 and 2.69, respectively.

### 4.2. Exploratory factor analysis

A varimax-rotated principal component analysis was employed to define orthogonal factors and extract their main indicators. We used the Kaiser–Meyer–Olkin Index (KMO = 0.87) and the Measure of Sampling Adequacy (MSA) for each indicator (Cerny and Kaiser, 1977) as sample adequacy criteria. In particular, based on the MSA measures, all indicators were above the 0.7 threshold index and were considered acceptable.

Table 5 shows that three factors with the criterion of an eigenvalue more significant than one were extracted. These represented 59.8% of the total variance, with loadings ranging from 0.45 to 0.86. Factor 1 represented the noise-*Sensitivity* of householders and explained 14.1% of the variances. Factor 2 showed high factors loading, principally for *pleasant* and then *calm* and *harmonious*. Factor 3 was measured by indicators including *attractive, liveable* and *safe*. Factors 2 and 3 explained 27.2 and 18.5% of the variance. The principal factors extracted from the EFA align with previous studies (Hong and Jeon, 2015). The corresponding structure was used in the CFA.

**Table 5:** Factor loadings for the analysis EFA model.

Indicators	MSA	Factor loading	Variance explained (%)
Factor 1: Noise-sensitivity			**14.1**
*I1. Easily awakened*	0.75	0.57	
*I2. Relaxation*	0.78	0.45	
*I3. Complain*	0.70	0.67	
*I4. Sensitivity*	0.71	0.65	
Factor 2: Sound-pleasantness			**27.2**
*I5. Pleasant*	0.87	0.86	
*I6. Calm*	0.91	0.78	
*I7. Harmonious*	0.87	0.86	
Factor 3: Visual-liveability			**18.5**
*I8. Attractive*	0.87	0.73	
*I9. Liveable*	0.86	0.79	
*I10. Safe*	0.94	0.57	

The bold values represent the total variance estimated for each factor.

### 4.3. Confirmatory factor analysis

A CFA (Long, 1983) was performed to examine the reliability and validity of the latent variables (see Table 6). A Cronbach-alpha coefficient with a minimum value of 0.7 is usually considered to determine good reliability (Nunnally and Bernstein, 1968). However, Hair et al. (2018) suggested examining convergent validity according to three reliability criteria values: construct reliability (CR), average variance extracted (AVE), and standardised factor loadings. The observed variables showed reasonably good convergent validity with values higher than 0.5 and confirmed satisfactory convergent validity (Std.factor.loading ≥0.5, AVE ≥ 0.6, CR ≥ 0.6). The constructs show good validity and adequate values of reliability, in line with recommended values in the literature (MSV < AVE, ASV < AVE; Zhao et al., 2021). Therefore, the goodness-of-fit estimates in the CFA model were barely accepted for the indicators associated with noise-*Sensitivity*, in contrast to the other factors of sound-*Pleasantness* and visual-*Liveability*, which were superior.

**Table 6:** CFA Results.

Latent variable	Cronbach’s alpha	Std. factor loading	CR	AVE	MSV	ASV
Noise-sensitivity $\eta_{1}$	**0.67**		**0.68**	**0.65**	**0.63**	**0.43**
*I1. Easily awakened*		0.56				
*I2. Relaxation*		0.50				
*I3. Complain*		0.64				
*I4. Sensitivity*		0.67				
Sound-pleasantness $\eta_{2}$	**0.94**		**0.94**	**0.84**	**0.30**	**0.18**
*I5. Pleasant*		0.94				
*I6. Calm*		0.89				
*I7. Harmonious*		0.91				
Visual-liveability $\eta_{3}$	**0.85**		**0.85**	**0.67**	**0.18**	**0.12**
*I8. Attractive*		0.81				
*I9. Liveable*		0.88				
*I10. Safe*		0.76				

The bold values represent the total variance estimated for each factor.

### 4.4. Concept of a structural equation model

Although there are studies examining associations between auditory and visual latent variables, we have not found prior theory-driven considerations to jointly explore the relationships between noise-*Sensitivity*, sound-*Pleasantness* and visual-*Liveability* (Aulia, 2016; Amir et al., 2021). Latent constructs, such as noise-*Sensitivity*, have been traditionally analysed jointly with person-related attributes (i.e., socio-demographic information) and noise-annoyance (Van Kamp et al., 2004).

**Figure 4:**
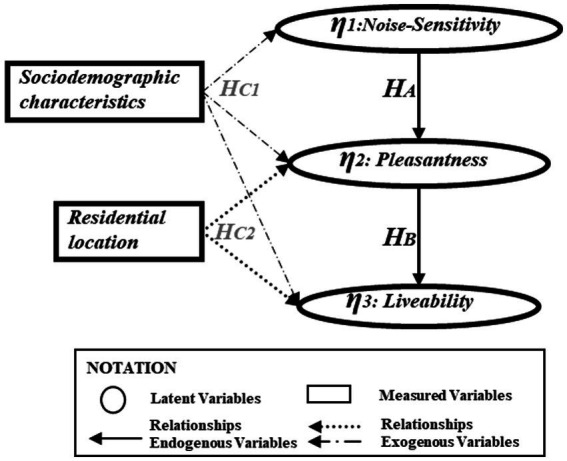
Conceptual SEM model.

Moreover, there is evidence in the literature to suggest that the socio-demographic variables influence both noise-*Sensitivity* and auditory factors and that these influences may change with location and activity (Hong et al., 2020; Tarlao et al., 2021). The effects of auditory and visual factors in different urban contexts have also been demonstrated. However, only a limited discussion is available concerning the influence of *Pleasantness* as an auditory factor and latent visual constructs such as *Liveability* (Van Kamp et al., 2003; Amir et al., 2021; Mouratidis and Yiannakou, 2022), especially in a Latin American context. Conversely, Yong et al. (2021) highlight that objective sound-visual components correlate with urban auditory factors. Within the framework of this paper, following relationships found in previous studies and former results, a conceptual SEM was tested concerning the four hypotheses depicted in Figure 4 for a residential environment context:

***H***_***A***_: The noise-Sensitivity of individuals negatively influences the Pleasantness of the sounds.

***H***_***B***_: The visual-Liveability conditions are positively correlated to sound-Pleasantness.

***H***_***C1*:**_ The socio-demographic characteristics of citizens influence noise-Sensitivity, sound-Pleasantness, and visual-Liveability.

***H***_***C2*:**_ The location of the dwelling influences noise-Sensitivity, sound-Pleasantness, and visual-Liveability.

The conceptual model in Figure 4 considers the socio-demographic and location variables as regressors and includes the latent variables. The values of the goodness-of-fit indices^2^ for the conceptual model are presented in Table 7. The socio-demographic variables were categorised as follows: *gender* (“female,” male), *age* (“young,” adult and elderly), *income level* (“low-income,” middle-income and upper-income), *education degree* (“elementary,” high-school and university), *employment status* (“unskilled,” qualified and specialised). The location variables were defined as *residential location* (“south-Quito,” north-Quito, and centre-Quito) and *land use* (“residential,” recreational, and commercial). All these variables have a reference category put in quotation marks (e.g., “south-Quito” is the reference category for residential locations in the city) to capture the heterogeneity across householders’ perceptions.

**Table 7:** Goodness-of-fit indices of the tested models and recommended values (*N* = 543).

Model fit index	$\chi_{SB}^{2}$ /df	CFI	TLI	RMSEA	SRMR
Conceptual model values	4.40	0.997	0.999	0.025	0.018
Modified model values	4.20	0.997	0.999	0.027	0.018
Recommended values	< 5.0	>0.90	>0.90	< 0.08	< 0.05

In particular, the respondent’s socio-demographics and residential location characteristics were associated with all latent constructs as explanatory variables. Besides, on the specification of *sound-Pleasantness* $\left(\eta_{2}\right)$ all acoustic components were also included (i.e., *L_Aeq,60s_*, *L_Ceq,60s_, L_10_*, *L_90_*, *L_10_*-*L_90,_ TSLV*). On the other hand, the understanding of *visual-Liveability* $\left(\eta_{3}\right)$ was complemented by the specification of the visual measures used in the configuration of the experimental design (see Table 4).

### 4.5. Modified structural equation model

**Figure 5:**
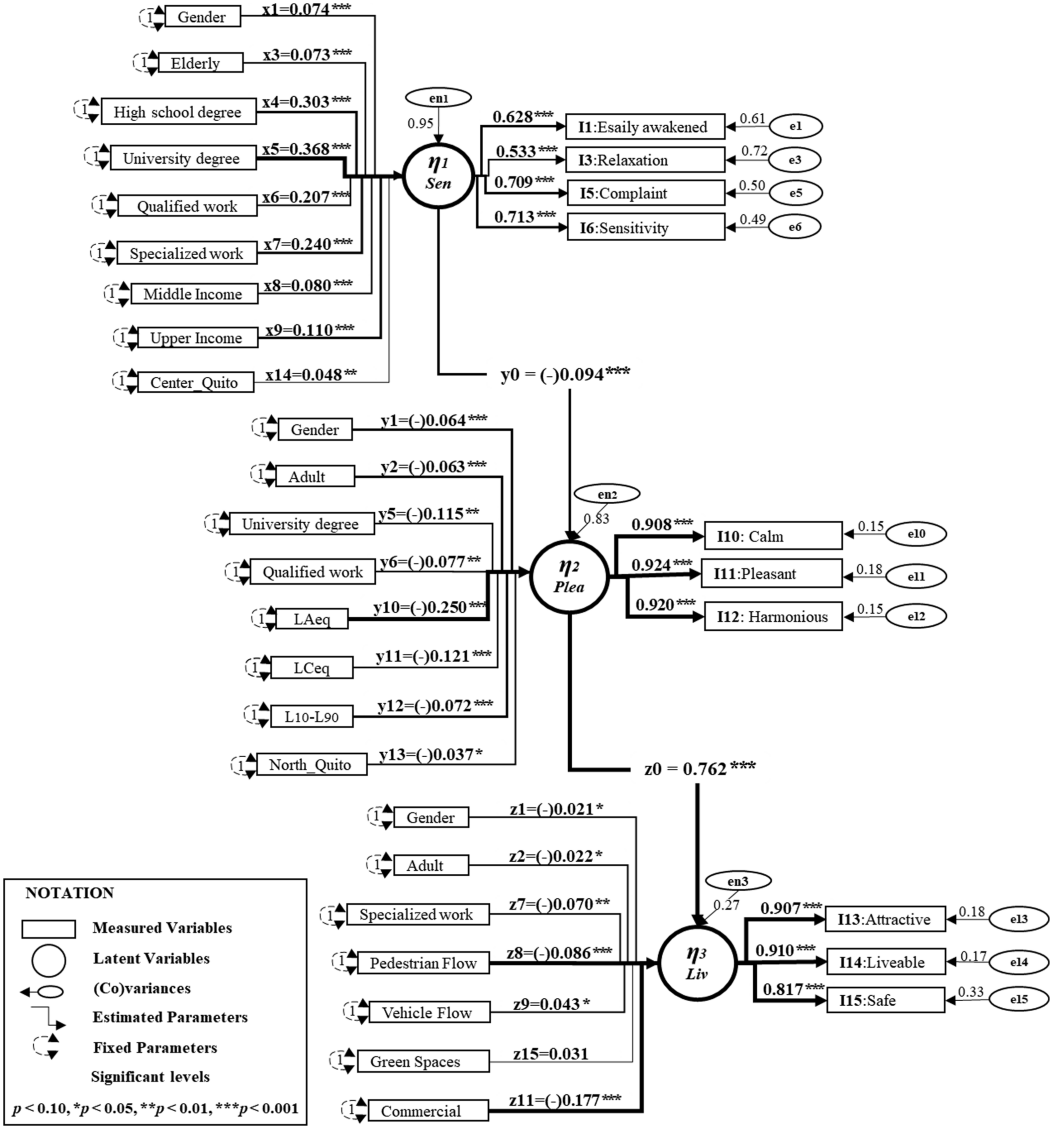
Results of the modified SEM model.

The approach to identifying the best path model was as follows. First, we checked if the regressors had the correct sign. Then, to define the paths, we specified those categories that were statistically significant. Finally, the modification process stopped when the goodness-of-fit indices surpassed the recommended values from the precedent model, as shown in Table 7. The modified model has 78 parameters, as shown in Figure 5, and the estimation results are given in Table 8. These results indicate a good-level-of-fit for assessing the structural model validity, according to the recommended criteria described in the literature (Hair et al., 2018). The regression path loadings of the models were estimated using the Diagonally Weighted Least Squares (DWLS) method, where the explanatory variables were specified as ordinal in the latent construct (Distefano and Morgan, 2014).

**Table 8:** Full structural equation model, measurement, and regressions.

Measurement	Estimate	S.E.	Value of *p*	Standardised estimate
Noise-sensitivity				
*I1. Easily awakened*	0.619	0.011	< 0.001	0.628
*I2. Relaxation*	0.523	0.012	< 0.001	0.533
*I3. Complaint*	0.701	0.011	< 0.001	0.709
*I4. Sensitivity*	0.704	0.011	< 0.001	0.713
Sound-pleasantness				
*I5. Pleasant*	0.907	0.003	< 0.001	0.924
*I6. Calm*	0.888	0.004	< 0.001	0.908
*I7. Harmonious*	0.902	0.003	< 0.001	0.920
Visual-liveability				
*I8. Attractive*	0.527	0.007	< 0.001	0.907
*I9. Liveable*	0.530	0.007	< 0.001	0.910
*I10. Safe*	0.464	0.006	< 0.001	0.817
Regressions	Noise-sensitivity	Sound-pleasantness	Visual-liveability	
Direct effect	(*y*_0_) −0.094^***^	(*z*_0_) 0.762^***^		
Gender	(*x*_1_) 0.074^***^	(*y_1_*) −0.064^***^	(*z*_1_) −0.021^*^	
Adult		(*y*_2_) −0.063^***^	(*z*_2_) −0.022^*^	
Elderly	(*x*_3_) 0.073^***^			
High-school-degree	(*x*_4_) 0.303^***^			
University-degree	(*x*_5_)0.368^***^	(*y*_5_) −0.115^**^		
Qualified-work	(*x*_6_) 0.207^***^	(*y*_6_) −0.077^**^		
Specialised-work	(*x*_7_) 0.240^***^		(*z*_7_) −0.070^**^	
Middle-income	(*x*_8_) 0.080^***^			
Upper-income	(*x*_9_) 0.110^***^			
Pedestrian flow			(*z*_8_) −0.086^***^	
Vehicles flow			(*z*_9_) 0.043^*^	
Green-spaces			(*z*_15_) 0.031	
Commercial land_use			(*z*_11_) −0.177^**^	
L_Aeq,60s_		(*y*_10_) −0.250^***^		
L_Ceq,60s_		(*y*_11_) −0.121^***^		
L_10_-L_90_		(*y*_12_) −0.072^***^		
North-Quito		(*y*_13_) −0.037^*^		
Centre-Quito	(*x*_14_) 0.048^**^			
Total effect				
Gender	(*x*_1_) 0.074^***^	(*y*_1_ + *y*_0_^*^x_1_) −0.071^***^	(*z*_1_ + *z*_0_^*^y_1_ + *z*_0_^*^y_0_^*^x_1_) −0.075^***^	
Adult		(*y*_2_) −0.063^***^	(*z*_2_ + *z*_0_^*^y_2_ + *z*_0_^*^y_0_^*^x_2_) −0.07^***^	
Elderly	(*x*_3_) 0.073^***^	(*y*_0_^*^x_3_) −0.007^***^	(*z*_0_^*^y_0_^*^x_3_) −0.005^***^	
High-school-degree	(*x*_4_) 0.303^***^	(*y*_0_^*^x_4_) −0.029^***^	(*z*_0_^*^y_0_^*^x_4_) −0.022^***^	
University-degree	(*x*_5_) 0.368^***^	(*y*_5_ + *y*_0_^*^x_5_) −0.150^***^	(*z*_0_^*^y_5_ + *z*_0_^*^y_0_^*^x_5_) −0.114^***^	
Qualified-work	(*x*_6_) 0.207^***^	(*y*_6_ + *y*_0_^*^x_6_) −0.097^***^	(*z*_0_^*^y_6_ + *z*_0_^*^y_0_^*^x_6_)−0.074^***^	
Specialised-work	(*x*_7_) 0.240^***^	(*y*_0_^*^x_7_) −0.023^***^	(*z*_7_ + *z*_0_^*^y_0_^*^x_7_) −0.087^**^	
Middle-income	(*x*_8_) 0.080^***^	(*y*_0_^*^x_8_) −0.008^***^	(*z*_0_^*^y_0_^*^x_8_) −0.006^**^	
Upper-income	(*x*_9_) 0.110^***^	(*y*_0_^*^x_9_) −0.010^***^	(*z*_0_^*^y_0_^*^x_9_) −0.008^***^	
Pedestrian flow			(*z*_8_) −0.086^***^	
Vehicle flow			(*z*_9_) 0.043^*^	
Green-spaces			(*z*_15_) 0.031	
Commercial land_use			(*z*_11_) −0.177^***^	
L_Aeq,60s_		(*y*_10_) −0.250^***^	(*z*_0_^*^y_10_) −0.019^***^	
L_Ceq,60s_		(*y*_11_) −0.121^***^	(*z*_0_^*^y_11_) −0.092^***^	
L_10_-L_90_		(*y*_12_) −0.072^***^	(*z*_0_^*^y_12_) −0.055^***^	
North-Quito		(*y*_13_) −0.037^*^	(*z*_0_^*^y_13_) −0.028^*^	
Centre-Quito	(*x*_14_) 0.048^**^	(*y*_0_^*^x_14_) −0.005^*^	(*z*_0_^*^y_0_^*^x_14_) −0.003^*^	

*p* < 0.10, ^*^p < 0.05, ^**^p < 0.01, ^***^p < 0.001. All effects are standardised. Significance levels are calculated with DWLS method.

### 4.6. Testing hypotheses H_A_ and H_B_

Table 8 shows the estimated parameters of the modified SEM model, such as standard error (S.E.), value of *p*, and standardised estimates. Regarding hypothesis H_A_, the standardised path loadings (*y*_0_ = −0.094) suggest that noise-*Sensitivity* negatively influences on sound-*Pleasantness* at the 95% confidence level. This result is consistent with those described by Tarlao et al. (2021). However, in this study, four indicators were used to measure noise-*Sensitivity*. The path underlying hypothesis H_B_ is accepted at the 95% confidence level, that is, there is a positive influence of sound-*Pleasantness* on visual-*Liveability* conditions (*z*_0_ = 0.762). To estimate the statistical effects of the structural equation model, we used reliable significance levels for the statistical effects, as shown below in Table 8.

Sound-*Pleasantness* (*η_2_*) was negatively correlated with the acoustic measurements of the A-weighted SPL (*y*_10_ = −0.25, *p* < 0.001), the equivalent C-weighted SPL (*y*_11_ = −0.121, *p* < 0.001) and the differences between percentiles L_10_–L_90_ (*y*_12_ = −0.072, *p* < 0.001). On the other hand, visual-*Liveability* (*η_3_*) was associated with decreased pedestrian flow (*z*_8_ = −0.086, *p* < 0.001). The visual components, such as vehicle flow (*z*_9_ = 0.043, *p* < 0.05) and green spaces (*z*_15_ = 0.031, *p* = 0.067), influenced positively visual-*Liveability* conditions. In contrast, the model revealed that being close to commercial zones (*z*_11_ = −0.177, *p* < 0.001) negatively affects people’s visual-*Liveability*.

### 4.7. Testing hypotheses H_C1_ and H_C2_ using a mediation analysis

Hypothesis H_C1_ was analysed considering the estimates of the total effect of the socio-economic variables on all perceptions *n_1_*, *n_2_* and *n_3_*. Hypothesis H_C2_ was tested regarding the total effect of the residential location on all factors *n*_*1*,_ *n*_*2*,_ and *n*_*3*._

Based on the stated hypotheses, we performed a mediation analysis by estimating the total effect between observed variables and the interrelated latent constructs. The total impact of each variable was computed by adding their direct and indirect effects (*z*_n_ + *z*_0_^*^y_n_ + *z*_0_^*^y_0_^*^x_n_). The indirect effect was obtained by multiplying the estimates of noise-*Sensitivity* (*y*_0_) or sound-*Pleasantness* (*z*_0_) and the reported direct effect of each categorised variable (*x*_n_, *y*_n_ and *z*_n_). Table 8 presents the total effects in an aggregated form, given the different directions of significant direct and indirect effects.

The mediation analysis reports a positive and significant correlation and total effect between *socio-economics* and noise-*Sensitivity*. Women tend to be more sensitive to noise than men (*x*_1_ = 0.074, *p* < 0.001). The increase in noise-*Sensitivity* in aged people is represented by the *elderly* variable (*x*_3_ = 0.073, *p* < 0.001). Noise-*Sensitivity* is also proportional to education level. The higher the education level, the higher the noise-*Sensitivity*. Heads of households with a level of education degree categorised as *high-school* (*x*_4_ = 0.303, *p* < 0.001) and *university-degree* (*x*_5_ = 0.368, *p* < 0.001) are more noise-sensitive than low educated participants. Following the same logic, noise-*Sensitivity* increases for those individuals with high-skilled jobs. Particularly, those participants performing *qualified-work* (*x*_6_ = 0.207, *p* < 0.001) and *specialised-work* (*x*_7_ = 0.240, *p* < 0.001) are more noise-sensitive than those in unskilled labours. Additionally, noise-*Sensitivity* increases for households reporting *middle-income* (*x*_8_ = 0.080, *p* < 0.001) and *upper-income* (*x*_9_ = 0.110, *p* < 0.001).

The mediation analysis also reports relationships between *socio-economics* and the sound-*Pleasantness* construct. The total effect was estimated by summing up the direct effect from socio-demographics interacting directly with the construct, and the indirect effect, which was determined by multiplying the (*y*_0_) value estimated for noise-*Sensitivity*. The analysis indicates a negative and significant correlation and total effect of *gender* (*y*_1_ + *y*_0_^*^x_1_ = −0.071, *p* < 0.001), *adult* (*y*_2_ *=* −0.063, *p* < 0.001), *elderly* (*y*_0_^*^x_3_ = −0.007, *p* < 0.001), *high-school-degree* (*y*_0_^*^x_4_ = −0.029, *p* < 0.001), *university-degree* (*y*_5_ + *y*_0_^*^x_5_ = −0.150, *p* < 0.01), *qualified-work* (*y*_6_ + *y*_0_^*^x_6_ = −0.097, *p* < 0.001), *specialised-work* (*y*_0_^*^x_7_ = −0.023, *p* < 0.001), *middle-income* (*y*_0_^*^x_8_ *=* −0.008, *p* < 0.001) and *upper-income* (*y*_0_^*^x_9_ *=* −0.010, *p* < 0.001). In other words, although women are more noise-sensitive, men find the sounds in residential environments less pleasant. Moreover, sound-*Pleasantness* in residential environments has an inverse relationship with *age*, *education level*, *employment* and *income* (i.e., sound-*Pleasantness* decreases when age increases).

The perception of the visual construct *Liveability* also varies across the population, according to several socio-demographic attributes. The indirect effect (*z*_n_ + *z*_0_^*^y_n_ + *z*_0_^*^y_0_^*^x_n_) was calculated by multiplying the estimates for the socio-demographics (*x*_n_, *y*_n_, *z*_n_) and the values (*z*_0_) and (*y*_0_) estimated for sound-*Pleasantness* and noise-*Sensitivity*, respectively. Men rated lower the construct of *Liveability* for the observed surroundings residential scenarios than women (z_1_ + z_0_^*^y_1_ + z_0_^*^y_0_^*^x_1_ = −0.075, *p* < 0.001). The results indicate that aged participants categorised such as *adult* (*z*_2_ + *z*_0_^*^y_2_ + z_0_^*^y_0_^*^x_2_ = −0.07, *p* < 0.001) and *elderly* (*z*_0_^*^y_0_^*^x_3_ = −0.005, *p* < 0.001) rated visual-*Liveability* conditions of the residential environment lower than younger residents. Additionally, visual-*Liveability* decreases in household heads with a higher educational degree. Particularly, those holding *high-school* (*z*_0_^*^y_0_^*^x_4_ = −0.022, *p* < 0.001) and *university* (*z*_0_^*^y_5_ + *z*_0_^*^y_0_^*^x_5_ = −0.114, *p* < 0.001) degrees reported lower *liveable* conditions than less educated participants. Visual-*Liveability* was also lower for those household heads performing *qualified* (*z*_0_^*^y_6_ + *z*_0_^*^y_0_^*^x_6_ = −0.074, *p* < 0.001) and *specialised* (*z*_7_+ *z*_0_^*^y_0_^*^x_7_ = −0.087, *p* < 0.01) work when compared with individuals performing unskilled activities. Finally, visual-*Liveability* perception decreases when income status increases. Specifically, *middle-income* (*z*_0_^*^y_0_^*^x_8_ = −0.006, *p* < 0.01) and *upper-income* (*z*_0_^*^y_0_^*^x_9_ = −0.008, *p* < 0.001) individuals reported lower *Liveability* conditions than low-income participants.

Therefore, considering the non-zero estimates of the total effects of *socio-economics* on the noise-*Sensitivity*, sound-*Pleasantness* and visual-*Liveability* constructs, we cannot reject the H_C1_ hypothesis at the 95% confidence level.

Furthermore, based on the mediation analysis and the relationships between the dwelling location of participants and the three latent constructs, the model suggests a significant heterogeneity effect amongst households placed in Quito’s main districts (North, Centre and South). Note that household heads living in *centre-Quito* (*x*_14_ = 0.048, *p* < 0.01) declared to be more sensitive to noise than those living in the city’s south. In addition, participants with a dwelling in *north-Quito* (*y*_13_ = −0.037, *p* < 0.05) and *centre-Quito* (*y*_0_^*^x_14_ = −0.005, *p* < 0.05) rated sound-*Pleasantness* lower than those located in *south-Quito*. Consequently, households living in *north-Quito* (*z*_0_^*^y_13_ = −0.028, *p* < 0.05) and *centre-Quito* (*z*_0_^*^y_0_^*^x_14_ = −0.003, *p* < 0.05) also rated lower visual***-**Liveability* than participants in *south-Quito*. This is probably because the city-centre and north areas of Quito have higher noise exposure than the southern part, due to increased commercial activities in those areas. The estimations of the total effects calculated above suggest that the perceptual dimensions vary according to the place of residence of the household heads, which allows accepting hypothesis H_C2_ at the 95% confidence level.

## 5. Discussion

This study provides new insights to identify the influence of household heads’ socio-demographic and housing location characteristics on their auditory and visual perceptions when selecting a residential environment. Previous knowledge especially that developed in the Latin American region, has provided limited evidence about the relationships between person-related attributes (i.e., noise annoyance), auditory and indirect visual factors in the residential context (Kogan et al., 2017; Rey-Gozalo et al., 2018). Our study also considers four relationships between auditory and visual perceptions in a residential setting. This is done by looking at the direct and indirect effects between objective attributes and the participants’ own personal traits.

The results of the SEM model show first, a significant negative relationship between noise sensitivity and sound pleasantness, and, indirectly, with visual liveability. This means that household heads who reported higher noise-*Sensitivity* also rated the sounds reproduced in the selected residential locations as less pleasant, in line with the result described by Tarlao et al. (2021). Also, participants are susceptible to a broader range of positive latent constructs and are not only associated with noise-annoyance as was found in previous studies (Fyhri and Klæboe, 2009; Ryu and Jeon, 2011). The examination of the direct influence of the objective sonic measures on sound-*Pleasantness* revealed a significant negative relationship with the values of the acoustic parameters L_Aeq,60s_, L_Ceq,60s_, and the percentile difference between L_A10_ and L_A90_. These influences could be compared with those recently described by Yong et al. (2021), except for the A-weighted SPL, and highlight the adequate association of sonic parameters on psychoacoustics descriptors as carriers of information when studying the enhancement of urban places.

Our study also demonstrates a second positive significant relationship between sound-*Pleasantness* and visual-*Liveability* in a Latin American context. Interestingly, in residential conditions, the direct effect of *Pedestrian flow* on visual-*Liveability* revealed a negative influence of this observable variable on the construct, differing from other studies where the presence of people in public spaces had been reported as a positive characteristic (Iglesias et al., 2013; Puyana-Romero et al., 2016), albeit from a safety point of view in the first case. It is possible that the negative sign was motivated by the restrictions on mobility during the COVID-19 pandemic, as people were warned to avoid crowds. Considering other visual components, we observed a positive relationship between *Vehicle-flow*, *Green spaces* and visual-*Liveability* in the residential context, and these results are in line with those described by Yong et al. (2021). In contrast, note that being close to *commercial* land use negatively influences visual-*Liveability*. In other words, the perception of *Liveability* differs amongst family heads because they are heterogeneous in terms of their socio-demographic and location attributes.

Based on the parameters estimated by the SEM model, which include a mediation analysis to assess direct effects and total effects loadings, our results reveal the relationships between socio-demographic characteristics of households heads and their perceptions of noise-*Sensitivity*, sound-*Pleasantness* and visual-*Livability*. The direct effect showed a negative influence between *socio-demographics* and noise-*Sensitivity*. Consistent with van Kamp et al. (2004), we found that the influence of socio-demographic characteristics on noise-*Sensitivity* varies significantly in the population under study. Family heads reported greater sensitivity if their education, employment and income status were higher. Thus, this study extends several patterns for noise-*Sensitivity* and auditory factors in a Latin American country. The direct effect estimates are influenced by age and gender. For example, respondents over 65 declared high sensitivity and perceived sounds as less pleasant than adults or young individuals. The model also suggests that older people rated sound-*Pleasantness* and visual-*Liveability* lower than younger people due to their noise-*Sensitivity*. Additionally, regarding *gender*, on average men in Quito perceive the residential environment as less pleasant and less liveable than women.

The last relationships concern the analysis of direct and total effects using the mediation analysis. Our results indicate that the household dwelling location and proximity to land uses and activities influence the latent constructs. This finding suggests a significant heterogeneity amongst households located in Quito’s main districts. For example, households located in the *centre* and *north* of the city reported a negative impact on both sound-*Pleasantness* and visual-*Liveability* compared to those living in the *south*. These findings suggest that families living in these zones could be affected by the higher sound pressure level exposure in those areas, especially for households located in the *centre* of Quito, which has much higher commercial activity. In this line, Arellana et al. (2019) have also argued that perceptions vary according to the residential location of the respondents.

## 6. Conclusion

This research provides evidence about the influence of explanatory variables such as auditory–visual components, socio-demographics and residential location characteristics on three latent constructs in a residential location context. Using the SEM model estimates and the mediation analysis, we found a negative correlation between noise-*Sensitivity* and sound-*Pleasantness*, and a positive relationship between sound-*Pleasantness* and visual-*Liveability*. Note that a limitation of this study is that we considered only sound-*Pleasantness* whilst keeping a parsimony criterion of the model structure. Future research should include eventfulness and pleasantness analysis in the Latin American context.

The findings reported in this paper complement the understanding of complex relationships described in the literature on the knowledge gap between the perception of sound-visual components with the added value of being assessed in the residential context of Latin American cities. Therefore, this study calls for a more holistic understanding of the perception of sound and visual attributes of residential environments rather than just independently assessing auditory perceptions, traditionally noise-annoyance, and other visual concepts associated with livability when studying improvements to the residential environment.

The estimations of parameters calculated from path regressions using SEM demonstrated the influences of the mediation analysis. The estimation of the direct effect showed that noise-*Sensitivity* correlates negatively with socio-demographics such as gender, age, education, employment and income status. The estimations calculated of the total effects provided a better understanding of when sound-*Pleasantness* mediates under the specification of the set of acoustic measures, such as the A-weighted SPL, the equivalent C-weighted SPL and percentiles L_10_–L_90_. Complementarily, estimates of the total effects calculated from the visual-*Liveability* path specification showed a heterogeneous variation across households according to their residential location in Quito’s main districts (North, Centre and South).

Our methodology adds evidence in favour of future research using digital surveys containing 360° video and immersive sound reproduction and the application of perceptual questionnaires to capture perceptions of audio-visual attributes of residential environments. This alternative approach to administrating the experiment stimulated participants’ senses with a high level of reality and offered portability advantages for conducting the survey. These improvements allowed participants to reproduce the experiment under home conditions and evaluate multiple residential environments. The overall flexibility of the implemented methodology provided adequate conditions for experimental replicability.

Our prior statistical estimations support the idea of complementing the study of audio-visual perceptions using advanced econometric theory. Therefore, the collected information on stated preferences is appropriate as an analytical tool for analysing residential location preferences considering sound and visual factors and attributes. Thus, using advanced hybrid discrete choice models, the willingness-to-pay measures are estimated considering environmental improvements, such as *noise reduction* or *enhanced urban amenities*. Hence, the authors of this study are currently working on this line of research.

## Data availability statement

The raw data supporting the conclusions of this article will be made available by the authors, without undue reservation.

## Ethics statement

Ethical review and approval was not required for the study involving human participants in accordance with the local legislation and institutional requirements. Written informed consent to participate in this study was not required from the participants in accordance with the national legislation and the institutional requirements.

## Author contributions

All authors listed have made a substantial, direct, and intellectual contribution to the work and approved it for publication.

## Conflict of interest

The authors declare that the research was conducted in the absence of any commercial or financial relationships that could be construed as a potential conflict of interest.

## Publisher’s note

All claims expressed in this article are solely those of the authors and do not necessarily represent those of their affiliated organizations, or those of the publisher, the editors and the reviewers. Any product that may be evaluated in this article, or claim that may be made by its manufacturer, is not guaranteed or endorsed by the publisher.

## Footnotes

^1^This refers to the streets conditions for trips measured by the vehicle level of service (LOS) type C defined in Transportation Research Board (2010) for road transport at the average speed range of 50 (mph) and measured in flow range (1,100 vehicles/h/lane) or equivalent (18 vehicles/min/lane).

^2^The chi-square statistic () is used for large and normalised sample sizes. CFI compares the difference in fit with a null model; CFI ≥ 0.95 is a good fit. TLI is the incremental fit index; TLI ≥ 0.90 is a good fit. The Root Mean Square Error approximation (RMSEA) privileges models with more degrees of freedom and larger samples; RMSEA ≤0.05 is a good fit. The Standardised Root Mean Square Residual (SRMR) measures the difference between implied and observed covariance matrices; SRMR ≤0.05 is a good fit.
